# Effectiveness of the Full Outline of UnResponsiveness Score in Patients With Acute Ischemic Stroke

**DOI:** 10.7759/cureus.22876

**Published:** 2022-03-05

**Authors:** Şeref Emre Atiş, Öner Bozan, Mehmet Esat Ferhatlar, Asim Kalkan

**Affiliations:** 1 Emergency Medicine, Karabük University Faculty of Medicine, Karabük, TUR; 2 Department of Emergency Medicine, Prof. Dr. Cemil Taşçıoğlu City Hospital, Istanbul, TUR; 3 Department of Emergency Medicine, Prof. Dr. Cemil Taşcıoğlu City Hospital, Istanbul, TUR

**Keywords:** intensive care unit, patient admission, glasgow coma scale, mortality, stroke

## Abstract

Background

Predicting the mortality and prognosis of patients with stroke is one of the commonly studied topics. Various scoring systems have been used in this regard. One of them is the Full Outline of UnResponsiveness (FOUR) score. In this study, we aimed to investigate the utility of the FOUR scores in terms of their ability to predict hospital stay duration and mortality in patients who were diagnosed with ischemic stroke upon their admission to the emergency department.

Methods

Our study is a prospective observational study. Patients who were admitted to the emergency department of a tertiary hospital and diagnosed with ischemic stroke between August 1, 2020, and August 1, 2021, were included in the study. The inclusion criteria were as follows: being over the age of 18, being diagnosed with ischemic stroke, having symptoms that started within the last 48 hours, and patient consent approved by the patients themselves or their relatives. The patients were divided into two groups according to the FOUR scores (FOUR score = 16 and FOUR score < 16). Patients’ demographic information, vital parameters, symptoms, time to admission, comorbidities, laboratory parameters, length of hospitalization, mortality, and Glasgow Coma Scale (GCS), FOUR, and National Institutes of Health Stroke Scale (NIHSS) scores were recorded.

Results

A total of 79 patients were included in the current study, of which 47 (59.5%) were male. The patients included in the present study had a mean age of 66 ± 13 years. When the two groups of patients with a FOUR score of 16 and a FOUR score of below 16 were compared, the mean platelet count was found to be 248 ± 70 × 10^3^/L in the former group and 170 ± 84 × 10^3^/L in the latter (p = 0.004). Sixty-five (91.5%) of the patients in the group with a FOUR score of 16 and three (37.5%) of the patients in the group with a FOUR score of less than 16 stayed for more than six hours in the hospital (p < 0.001). When the patients were evaluated for intensive care unit (ICU) admission rates, five (62.5%) patients with a FOUR score of <16 were admitted to the ICU. This rate was 2.8% (n = 2) in the group of patients with a FOUR score of 16 and was found to be significantly lower (p < 0.001).

Conclusion

The FOUR score was found to be useful in predicting the ICU admission rate of patients with ischemic stroke. It has also been shown that the admission time was shorter in patients with a lower FOUR score, and platelet counts were also lower in this group.

## Introduction

Stroke is the second most common cause of death worldwide, following heart disease [1]. Predicting the mortality and prognosis of patients with stroke is one of the commonly studied topics [2]. The Glasgow Coma Scale (GCS), which is widely used to predict mortality and prognosis, evaluates the following: eye-opening, verbal, and motor responses [3]. However, in patients with speech disorders, GCS-based evaluations may sometimes lead to false predictions since the verbal response of these patients is unreliable [4].

The Full Outline of UnResponsiveness (FOUR) score, on the other hand, is a scale that evaluates eye response, motor response, brainstem reflexes, and respiration patterns on a scale of 0-4 [5]. Since the FOUR score evaluates brainstem reflexes and respiration patterns instead of verbal responses, it is thought to compensate for what the GCS does not offer [6]. Within this context, the efficiency of the FOUR score and GCS in predicting the prognosis of patients with diseases such as meningitis and trauma has been compared [7]. There are very few studies comparing the GCS and FOUR score in terms of their mortality and prognosis predictive values in patients with ischemic stroke admitted to the emergency department [8,9].

In this study, we aimed to compare the GCS and FOUR score in terms of their ability to predict hospital stay duration and mortality in patients who were diagnosed with ischemic stroke upon their admission to the emergency department.

## Materials and methods

Study design

Our study is a prospective observational study. It was conducted after the approval of Prof. Dr. Cemil Taşçıoğlu City Hospital ethics committee was obtained (approval number: 48670771-514.10/324). Additionally, the study was conducted in accordance with the Declaration of Helsinki, and informed consent forms were collected from the patients included in the study.

Patient selection

Our study included patients who were admitted to the emergency department of a tertiary hospital and diagnosed with ischemic stroke between August 1, 2020, and August 1, 2021. The inclusion criteria were as follows: being over the age of 18, being diagnosed with ischemic stroke, having symptoms that started within the last 48 hours, and patient consent approved by the patients themselves or their relatives. Patients having neuropsychiatric or neurodegenerative diseases such as schizophrenia and Parkinson’s disease, pregnant women, patients with lesions in the central nervous system, and those who used drugs affecting primarily the central nervous system (such as antidepressants and antipsychotics) were excluded from the study.

Data collection

Study data included patients’ demographic information such as age and gender; vital status (arterial blood pressure, pulse, and oxygen saturation); symptoms (lateralization, speech impairment, and others); time to admission; comorbidities; white blood cell and platelet counts; hemoglobin, sodium, potassium, urea, creatinine, glucose, alanine transferase, aspartate transferase, and C-reactive protein values; length of hospitalization; and GCS, FOUR, and National Institutes of Health Stroke Scale (NIHSS) scores. The short-term (28-day) mortality of the patients was also recorded. The patients were divided into two groups according to the FOUR scores (FOUR score = 16 and FOUR score < 16).

Primary outcome

Our primary objective was to investigate the adequacy of the FOUR score in predicting the length of hospital stay, prognosis, and mortality of patients with stroke.

Statistical analysis

To analyze the findings obtained in this study, the IBM SPSS version 22 (IBM Corporation, Armonk, NY, USA) software was used. The normality distribution of the parameters was evaluated using the Shapiro-Wilk test, Q-Q plots, and their median and mean n values. In addition to the descriptive statistical methods (mean, standard deviation (SD), median and interquartile ranges, and frequency), the Mann-Whitney U test was performed for the parameters where the compared quantitative data did not conform to a normal distribution, and the Student’s t-test was performed for the parameters that conformed to normal distribution. The chi-square test was used to compare qualitative data, and Fisher’s exact test was used for the parameters with values below the expected count. Pearson’s correlation analysis was employed to evaluate the relationship between the two scoring scales. p < 0.05 was considered statistically significant.

## Results

A total of 79 patients were included in the current study, of which 47 (59.5%) were male. The patients included in the present study had a mean age of 66 ± 13 years. The number of patients with a FOUR score of 16 was 71 (89.9%). When the patients were examined for the presence of additional diseases, hypertension was detected in 54 (68.4%) patients, and ischemic heart disease was detected in 12 (15.2%). When they were evaluated for complaints during admission, it was understood that 30 (38.0%) patients were admitted with lateralization. The patients had a median length of hospitalization of six (5-7) days. Other demographic data, additional diseases, hospital stay duration, and patient outcomes are summarized in Table 1.

**Table 1:** Patients’ demographic data, comorbidities, complaints, length of hospitalization and admission, and outcomes

Parameter	Value
Age (mean ± SD)	66 ± 13
Gender	
Female (n (%))	32 (40.5%)
Male (n (%))	47 (59.5%)
Complaint	
Lateralization (n (%))	30 (38%)
Speech impairment (n (%))	19 (24%)
Other (n (%))	30 (38%)
Time to admission (hour)	
Less than three hours (n (%))	10 (12.7%)
Three to six hours (n (%))	1 (1.3%)
More than six hours (n (%))	68 (86%)
Length of hospitalization (day) (median–interquartile, 25–75)	6 (5–7)
Hypertension (n (%))	54 (68.4%)
Diabetes mellitus (n (%))	27 (34.2%)
Ischemic heart disease (n (%))	12 (15.2%)
Chronic renal failure (n (%))	6 (7.6%)
Chronic obstructive pulmonary disease (n (%))	3 (3.8%)
Cerebrovascular event (n (%))	18 (22.8%)
Malignancy (n (%))	4 (5.1%)
Other neurologic disorders (n (%))	3 (3.8%)
ICU admission	
No (n (%))	72 (91.1%)
Yes (n (%))	7 (8.9%)
Mortality (n (%))	3 (3.8%)

When the two groups of patients with FOUR score of 16 and FOUR score of below 16 were compared, the mean platelet count was found to be 248 ± 70 × 10^3^/L in the former group and 170 ± 84 × 10^3^/L in the latter. The platelet count of the group of patients with a FOUR score of <16 was significantly lower than that of the other group (p = 0.004). There was no significant difference between the groups in terms of vital status or other laboratory parameters (Table 2).

**Table 2:** Comparison of vital parameters and laboratory values according to FOUR score groups * Parameters with normal distribution were compared using the Student’s t-test. ^χ^ Chi-square and Fisher’s exact tests were used to compare nominal data. ^¥^ Parameters without normal distribution were compared using the Mann–Whitney U test. BP: blood pressure

	FOUR score = 16 (n = 71)	FOUR score < 16 (n = 8)	Total	p
Age	65 ± 12	72 ± 18	66 ± 13	0.165^*^
Gender (male)	40 (56.3%)	7 (87.5%)	47 (59.5%)	0.133^χ^
Systolic BP (mmHg)	160 ± 31	140 ± 40	158 ± 32	0.085
Diastolic BP (mmHg)	86 ± 16	76 ± 12	85 ± 20	0.114
Pulse (per minute)	82 ± 14	77 ± 12	82 ± 13	0.899
SpO₂ (%)	97 ± 2	97 ± 1	97 ± 2	0.966
WBC (10^3^/L)	8.127 ± 3.320	8.229 ± 3.487	8.137 ± 3.314	0.935
Hemoglobin (g/dL)	14.65 ± 10.07	12.41 ± 2.40	14.42 ± 9.59	0.535
Platelet (10^3^/L)	248 ± 70	170 ± 84	240 ± 75	0.004
Sodium (mmol/L)	138 ± 3	139 ± 3	138 ± 3	0.323
Potassium (mmol/L)	4 ± 0.5	4 ± 0.3	4 ± 0.5	0.322
Glucose (mg/dL)	125 (179–102)	152 (107–205)	125 (103–181)	0.440^¥^
Urea (mg/dL)	38 (30–49)	47 (30–53)	39 (30–49)	0.407
Creatinine (mg/dL)	0.89 (0.71–1.07)	0.99 (0.74–1.48)	0.90 (0.71–1.08)	0.363
ALT (U/L)	17 (13–24)	20 (6–51)	17 (13–24)	0.916
AST (U/L)	21 (17–27)	21 (15–41)	21 (17–27)	0.909
CRP (mg/L)	6.40 (3.07–14.10)	3.13 (1.15–8.41)	6.00 (2.71–13.50)	0.141

When the admission times to the hospital were examined, 65 (91.5%) of the patients in the group with a FOUR score of 16 and three (37.5%) of the patients in the group with a FOUR score less than 16 stayed for more than six hours in the hospital (p < 0.001). Moreover, the median GCS score of the group of patients with a FOUR score of <16 was found to be 11 (10-15), which was lower than that of the group of patients with a FOUR score of 16 (p = 0.003). When the patients were evaluated for intensive care unit (ICU) admission rates, five (62.5%) patients with a FOUR score of <16 were admitted to the ICU. This rate was 2.8% (n = 2) in the group of patients with a FOUR score of 16 and was found to be significantly lower (p < 0.001). The comparisons of patient complaints, hospitalization duration, and NIHSS values are summarized in Table 3.

**Table 3:** Comparison of complaints, time to admission, length of hospitalization, admission to ICU, and GCS and NIHSS scores according to FOUR score groups ^χ^ Chi-square and Fisher’s exact tests were used to compare nominal data. ^¥^ Parameters without normal distribution were compared using the Mann–Whitney U test. ICU: intensive care unit, GCS: Glasgow Coma Scale, NIHSS: National Institutes of Health Stroke Scale

	FOUR score = 16 (n = 71)	FOUR score < 16 (n = 8)	p
Complaint (lateralization)	27 (38%)	3 (37.5%)	0.587^ χ^
Time to admission (more than six hours)	65 (91.5%)	3 (37.5%)	<0.001
Admission to ICU (yes)	2 (2.8%)	5 (62.5%)	<0.001
Length of hospitalization	6 (5–7)	8 (4–18)	0.193^¥^
GCS	15 (15–12)	11 (10–15)	0.003
NIHSS	4 (3–6)	7 (3–13)	0.114

When studied in terms of its relationship with the other two scoring systems, i.e., the GCS and the NIHSS, the FOUR score was found to have a slightly significant positive correlation with the GCS score (0.329, p < 0.01) and not to have a significant correlation with the NIHSS score (-0.179, p > 0.05). There was also a moderately significant negative correlation between the NIHSS and GCS scores (-0.503, p < 0.01) (Table 4).

**Table 4:** Correlation analysis between FOUR score and other scorings Pearson correlation analysis ** p < 0.01 FOUR: Full Outline of UnResponsiveness, GCS: Glasgow Coma Scale, NIHSS: National Institutes of Health Stroke Scale

		FOUR score	GCS	NIHSS
FOUR score	r	1	0.329^**^	-0.179
GCS	r	0.329^**^	1	-0.503^**^
NIHSS	r	-0.179	-0.503^**^	1

## Discussion

In our study, the group of patients with a FOUR score of <16 had lower platelet counts than the group of patients with a FOUR score of 16. In a meta-analysis conducted by Sadeghi et al. [10], platelet counts were found to be lower in the group of patients with acute ischemic stroke than in the control group. In another study, low platelet counts were identified as a predictor of in-hospital mortality rate [11].

The FOUR and GCS scores were found to be independent predictors of mortality in patients with bacterial meningitis, and these two scoring systems were found to be strongly correlated to each other [12]. In a study where Bayraktar et al. [13] compared the FOUR scores with the GCS scores in relation to the mortality of patients followed up in the ICU, the FOUR score was reported to be more effective than the GCS in predicting mortality [14-15]. There are studies reporting the FOUR score to be effective in predicting prognosis especially in patients with stroke since, unlike the GCS, the FOUR score is not dependent on verbal responses and provides insight into brainstem reflexes [2]. Studies focusing on emergency room populations demonstrated that, compared to the GCS, the FOUR score had an equal or superior prognostic value in predicting mortality [16]. Similar to the literature, our study also found that ICU admission rates were higher in the patients with a FOUR score of <16 than those with a FOUR score of 16. Additionally, the patients with a FOUR score of <16 had lower GCS scores and stayed in the hospital for a shorter time. However, due to the relatively small number of patients included in the study and the small number of patients (three patients) developing mortality, it was not possible to estimate mortality.

In the study, the FOUR score was compared not only with the GCS score but also with the NIHSS score. While the FOUR score had a slightly significant statistical correlation with the GCS, there was no statistically significant relationship between the FOUR score and the NIHSS. Similar to our study, other studies demonstrated that there was a correlation between the GCS and the FOUR score [12]. Moreover, unlike our study, there are some studies that show a strong correlation between the FOUR score and the NIHSS [4]. The relatively small number of patients included herein might be the reason why the present study has not found a correlation between the NIHSS and the FOUR score. We believe that the relationship between these two scoring systems can be established in further studies that recruit a larger patient population.

Limitations

This was a single-center study, and the number of included patients was relatively small. Additionally, the present study did not investigate the evaluation consistency between the emergency medicine doctors who evaluated the scores that are compared herein. There is no such evaluation method wherein the scores are compared by doctors belonging to multiple medical fields and where such doctors are evaluated for the consistency they might or might not have within their own field. Due to the relatively small number of patients included in the study, it was not possible to estimate mortality.

## Conclusions

The FOUR score was found to be useful in predicting the ICU admission rate of patients with ischemic stroke. It has also been shown that the admission time was shorter in patients with lower FOUR scores, and platelet counts were also lower in this group. However, no correlation was found between the demographic data, vital parameters, and laboratory values ​​of the patients and the FOUR score.

The FOUR score has a positive correlation with the Glasgow Coma Scale, but not with the National Institutes of Health Stroke Scale. There is a negative relationship between the Glasgow Coma Scale and the National Institutes of Health Stroke Scale.
